# Research agenda evaluating measurement-based care for opioid use disorder among patients with co-occurring depressive disorders

**DOI:** 10.3389/fpsyt.2025.1624642

**Published:** 2025-08-07

**Authors:** Udi E. Ghitza

**Affiliations:** Center for the Clinical Trials Network (CCTN), National Institute on Drug Abuse (NIDA), National Institutes of Health (NIH), US Department of Health and Human Services (HHS), Bethesda, MD, United States

**Keywords:** opioid use disorder, vulnerable populations, tailored interventions, substance use, treatment, depressive disorders, depression, mood disorders

## 1 Introduction

Buprenorphine and other medication treatments for opioid use disorder (OUD) in general medical settings are effective in preventing drug overdoses related to opioid use, although treatment retention is often challenging. Real-world data indicate high medication discontinuation rates at 6 months or greater following treatment initiation, partially indicative of a common failure to optimize medication dosing to minimize side effects, maximize therapeutic effects, and sustain treatment engagement and adherence (1, 2). These barriers hinder the achievement of optimal clinical outcomes in managing conditions like OUD, which is often a chronic relapsing condition and frequently associated with mood disorders. Measurement-based care (MBC) may be defined as an evidence-based healthcare approach in which systematic outcome monitoring of disease severity and symptomatology over time yields actionable feedback to providers to guide their clinical decision-making on how to customize medication dosing promptly to improve patients’ treatment outcomes.

## 2 Research agenda evaluating measurement-based care for opioid use disorder among patients with co-occurring depressive disorders

MBC clinical trials in substance use disorders are in their early stages, particularly in vulnerable individuals with co-occurring mood disorders. Pragmatic implementation research is needed to determine how stepped care management of medication treatments for OUD (MOUD) utilizing MBC can be effectively operationalized to enhance symptom control, improve overall functioning, and increase treatment retention, compared to conventional treatment approaches in community-based programs and office-based MOUD settings. MBC relies on brief, validated measures assessing clinically relevant parameters related to OUD. These parameters encompass the level of opioid use, withdrawal symptom severity, craving, the duration and severity of subjective effects when opioids are used, and their side effects. Validated brief assessments provide ratings for these parameters to guide providers’ decision-making. Some examples include, but are not limited to, the 7-item PROMIS-based Opioid Use Monitor (OUM) to evaluate these clinically meaningful parameters, and core OUD signs and symptoms based upon the five DSM-5 OUD domains from the Marsden et al. (2019) measure to assess treatment outcomes. Such ratings inform providers to make necessary adjustments to medication regimens and dosing when treatment outcomes fall short (3, 4).

These assessments can be deployed to create actionable clinical work plans where providers adjust medication dosing guided by MBC feedback, followed by re-evaluating clinical effectiveness through regular, repeated measurement over time after making titration adjustments. MBC is often conducted through a shared decision-making process between patients and their providers, fostering an environment of patient coaching and problem-solving. This approach equips patients to self-manage their condition actively, to engage in clinical decision-making, and to adopt health-promoting behaviors that enhance overall wellness, treatment retention, and recovery in chronic disease management (3, 4).

In people addicted to opioids, depressive symptomatology may co-occur with prolonged opioid withdrawal and complicate recovery, mediated by extended amygdala neurocircuitry allostatic changes (5, 6). When individuals with OUD become addicted and are less sensitive to the pleasurable effects of opioids over time, they tend to show intolerance to stressors and are more sensitive to aversive symptoms of opioid withdrawal, which increase the likelihood for relapses to temporarily relieve this negative emotional state (5, 6). Until recently, illicit opioid use in the United States has been on the rise for much of the decade since 2015, along with increases in opioid-related overdose deaths (7). There has been a parallel increase in rates of depressive disorders in the United States over this timeframe (8). A significant contributor to rising mortality in the United States for much of the last decade includes deaths of despair or fatalities from suicide and drug overdoses related to synthetic opioids such as fentanyl (9). Treatment-seeking patients with OUD tend to display a greater prevalence of comorbidity with depressive disorders relative to the general population in the United States, with approximately 42 percent of these individuals with OUD also reporting depressive disorders (10). Research suggests that untreated mood disorders among patients with substance use disorders are associated with worse treatment outcomes and prognosis (11, 12). If patients do not achieve desired treatment goals with OUD medication treatments and they display symptomatology indicating untreated unipolar depression, evidence-based MBC strategies already well-established for depression care management can be conducted to support chronic disease management among these vulnerable individuals. Validated measures to monitor symptoms of depression and promptly inform treatment decisions include the 9-item version of the Patient Health Questionnaire (PHQ-9), 16-item Quick Inventory of Depressive-Symptomatology-Self-Report (QIDS-SR_16_), or other validated assessments. These measures are paired with practice guidelines for the treatment of patients with depressive disorders. Accompanying actionable clinical decision support tools use these assessment scores to guide the selection and the switching or augmenting of antidepressant medications, monitoring adverse events and treatment adherence, reviewing comorbidities, and adjusting the treatment plan, as needed, to maximize the likelihood of recovery success. In summary, MBC may be performed to personalize dosing modification procedures based upon regular measurement of symptoms and side effects to maintain sufficient antidepressant doses or recalibrate these, if needed, to optimize treatment outcomes (13–17).

Healthcare data science research using electronic health records data from patients in various health systems suggests that several antidepressants may be promising to improve OUD treatment outcomes. Zhou et al. (2021) conducted an extensive retrospective case-control study using de-identified electronic health records data to assess repurposed candidate medications for treating OUD. They found that three antidepressants (bupropion, atomoxetine, and mirtazapine) were associated with odds of OUD remission nearly 50% higher relative to patients with depression who were not prescribed these antidepressants (18). Using these candidate medications for treating OUD in an adaptive design manner and a stepped care management approach, patients may be offered any one of these antidepressants as adjuncts to MOUD, depending on how well treatment goals and remission success are achieved with them. An adaptive design care management approach may be warranted since up to 20% of depressed patients are treatment-resistant (19). If treatment-outcome endpoints are unmet at critical clinical decision points, information provided through MBC could inform switching to another of these antidepressants. This MBC approach may be evaluated for effectiveness to increase the likelihood of remission from OUD and depressive disorders, and for implementation sustainability in pragmatic, double-blind, randomized-controlled trials attending to vulnerable patient populations exhibiting co-occurring depressive symptomatology.

## 3 Discussion

Within the context of measurement-based care, a gap need area is multisite effectiveness-implementation clinical trials research to be conducted utilizing patient-centered care approaches incorporating shared decision-making procedures as a springboard for collaborative patient-provider discussions attending to personalized patient preferences for treatment options, with interventions matched to symptoms. These collaborative discussions should take into account (a) OUD severity, aforementioned OUD-specific parameters that impact prognosis, and the current stage along the addiction disease process, (b) the presence of other substance use disorders, co-occurring psychiatric and other medical conditions which may lower the likelihood of remission success, (c) motivation of individuals to reduce their medically harmful opioid use, (d) reasons for hesitancy or readiness to do so, (e) coaching patients to self-manage their conditions and in clinical decision making—reinforcing problem-solving skills to facilitate health-promoting behaviors which strengthen resiliency to stress, and (f) how to assist patients in implementing a treatment plan consistent with their readiness to change, their preferences, co-occurring conditions, and addressing self-reported prominent barriers to their recovery. Endpoints of interest may relate to addressing key gaps in the opioid care cascade, including sustaining patient engagement in care, retention in treatment, and enhancing OUD remission and abstinence from illicit opioid use, life satisfaction, psychosocial functioning, and community involvement, as well as the quality of life throughout patients’ recovery.

The National Institute on Drug Abuse (NIDA) Clinical Trials Network (CTN) and similar clinical research enterprises may be potential venues to propose conducting this research agenda. Since its founding in 1999, the mission of CTN has been to bridge the gap between research and clinical practice by rapidly translating science into evidence-based care for treating substance use disorders. CTN has more than 25 years of experience and investigative teams’ expertise performing rigorous multisite randomized-controlled trials on pharmacotherapeutic, behavioral, and integrated treatments to test their effectiveness among US general medical settings, community-based treatment programs, and various patient populations. An understudied gap needs area worthy of consideration remains developing, validating, and refining for clinical practice sustainability those evidence-based treatment approaches to chronic disease management, such as MBC, serving vulnerable and underserved OUD patients with untreated co-occurring depressive disorders.
